# Traditional Machine and Deep Learning for Predicting Toxicity Endpoints

**DOI:** 10.3390/molecules28010217

**Published:** 2022-12-26

**Authors:** Ulf Norinder

**Affiliations:** Department of Computer and Systems Sciences, Stockholm University, 164 07 Kista, Sweden; ulfn@dsv.su.se

**Keywords:** CATMoS dataset, CDDD, BERT, conformal prediction, random forest, RDKit

## Abstract

Molecular structure property modeling is an increasingly important tool for predicting compounds with desired properties due to the expensive and resource-intensive nature and the problem of toxicity-related attrition in late phases during drug discovery and development. Lately, the interest for applying deep learning techniques has increased considerably. This investigation compares the traditional physico-chemical descriptor and machine learning-based approaches through autoencoder generated descriptors to two different descriptor-free, Simplified Molecular Input Line Entry System (SMILES) based, deep learning architectures of Bidirectional Encoder Representations from Transformers (BERT) type using the Mondrian aggregated conformal prediction method as overarching framework. The results show for the binary CATMoS non-toxic and very-toxic datasets that for the former, almost equally balanced, dataset all methods perform equally well while for the latter dataset, with an 11-fold difference between the two classes, the MolBERT model based on a large pre-trained network performs somewhat better compared to the rest with high efficiency for both classes (0.93–0.94) as well as high values for sensitivity, specificity and balanced accuracy (0.86–0.87). The descriptor-free, SMILES-based, deep learning BERT architectures seem capable of producing well-balanced predictive models with defined applicability domains. This work also demonstrates that the class imbalance problem is gracefully handled through the use of Mondrian conformal prediction without the use of over- and/or under-sampling, weighting of classes or cost-sensitive methods.

## 1. Introduction

Drug discovery is an expensive and resource-intensive process that involves many challenges, not least within the area of toxicity [1]. A growing concern in drug development is the high attrition rate in late clinic trials due to issues related to toxicity [2]. Computational methods, e.g., computer-aided drug design, have thus become standard tools in order to improve the efficiency of the drug discovery process and to mitigate undesirable toxicity effects [3,4,5,6].

Methods such as molecular structure property modeling have been used for decades in the pharmaceutical field in order to predict important properties, e.g., biological activity, solubility and toxicity, for prioritization of compounds with respect to potential toxicity issues and experimental testing [7]. For a recent review on computational tools, see reference [8]. The increasing focus on identifying undesirable toxic effects for chemical structures of interest, real or virtual, is manifested by recent publications such as [9,10] and recent reviews on machine learning (ML) techniques by Dara and co-workers [11] and by Matsuzaka and Yashiro [12].

During the last few years, deep learning (DL) techniques have successfully been applied in various domains, e.g., natural language processing [13] as well as image analysis [14], and have increased the interest for applying such methodologies also for molecular property predictions [15].

Many of the DL investigations have used the Simplified Molecular Input Line Entry System (SMILES) notation [16] as a starting point for feature generation and have shown impressive performances [17,18,19,20]. Several different architectures have been investigated such as autoencoders [18], convolutional neural networks [21], recurrent neural networks [22] and transformers [23,24,25]. Lately, publications using different implementations of Bidirectional Encoder Representations from Transformers (BERT) have been published [26,27].

The Mistra SafeChem research program, financed by Mistra (The Swedish Foundation for Strategic Environmental Research, Stockholm, Sweden), has the overarching aims to create a sustainable chemical industry and reduce exposure to hazardous substances [28]. One activity, among many other objectives and activities, is to develop and use *in silico* predictive models for early prediction and verification of hazardous properties of new molecules or materials. This includes the development of molecular property models for predicting toxicity, e.g., acute toxicity.

The well-known CATMoS benchmark dataset [29] has been used in this study for modeling acute toxicity and the results from both traditional machine learning as well as deep leaning are presented.

## 2. Materials and Methods

### 2.1. CATMoS Datasets

The dataset can be downloaded from reference [29].

The originally defined training and evaluation set in [29] were used.

The training, validation and conformal prediction (CP) calibration sets were randomly selected from the original training set.

Two different binary classification sets were investigated:

The very toxic (VT; catmos_vt) and the non-toxic (NT; catmos_nt).

Table 1 shows the number of compounds in each set after standardization.

### 2.2. Feature Generation

#### 2.2.1. Structure Standardization

The structures, represented as SMILES, were standardized using the “remove_salt_stereo” and “organic_filter” functions of the “preprocessing.py” script found in the Continuous and Data-Driven Descriptors (CDDD) GitHub repository [30], neutralized, followed by RDKiT smiles tautomer standardization [31]. Additional structures were excluded due to MolBERT sequence length errors.

#### 2.2.2. RDKit Descriptors

A total of 96 different physiochemical descriptors were calculated using MolecularDescriptorCalculator in RDKit [31] (list of calculated descriptors is available in Supplementary Materials S2).

#### 2.2.3. CDDD Descriptors

In total, 512 CDDD descriptors of length 512 were calculated using the CDDD GitHub repository code and model [30]. The RNN architecture-based translation model translating a SMILES string into its canonical SMILES was used.

### 2.3. Traditional Machine Learning

The binary classification models using RKDit and CDDD descriptors, respectively, were built using the Scikitlearn RandomForestClassifier [32] with default options (as part of the conformal prediction model generation, see Section 2.5 for details) [31].

### 2.4. Deep Learning

Two different approaches were used for generating BERT models.

#### 2.4.1. MolBERT

The MolBERT binary classification models were fine-tuned using the MolBERT GitHub repository code [33] and the pre-trained model available at reference [34]. The fine-tuning network consisted of a single linear layer connected to the pooled transformer output. Default settings were used for fine-tuning. A validation set was used to avoid over-fitting of the model as well as check-pointing for saving the “best” model according to the validation set. Ten models were built using different initialization seeds.

A second set of models where fine-tuned using a pre-trained model on 500 k randomly selected PubChem compounds with a validation set of 50 k compounds.

#### 2.4.2. Molecular-Graph-BERT

The Molecular-graph-BERT binary classification models were fine-tuned using the Molecular-graph-BERT GitHub repository code [35] and a Molecular-graph-BERT pre-trained 500 k PubChem model. The fine-tuning network consisted of a two-layer fully connected neural network attached to the transformer encoder layer output. Default settings were used for fine-tuning. A validation set was used to avoid over-fitting of the model as well as check-pointing for saving the “best” model according to the validation set. Ten models were built using different initialization seeds.

### 2.5. Conformal Prediction

A conformal predictor (CP) is a member of a family called confidence predictors [36]. These predictors have several useful properties for prediction tasks in biomedical research [37]. A particularly useful property of conformal prediction is that the method will result in valid predictions based on a user-defined significance level, i.e., a level of acceptable percentage of errors, given that the data is exchangeable. This property of validity is based on a mathematical proof by Volk and co-workers [36]. In this investigation, we used Mondrian (inductive) conformal prediction that guarantees validity for each of the two classes independently and finally median aggregated the conformal prediction outcomes from the 10 developed models for each compound and each class for final class assignment [38].

The nonconformist package [39], where scikit-learn algorithms such as the RandomForestClassifier serve as a base classifier, was employed that provide the results from conformal prediction. The following expression was used in the ICP function (Condition = lambda x: x [1]) in order to enable Mondrian conformal prediction in the nonconformist package.

An in-house script was employed to perform the conversion of predictions (scoring) from the models using BERT algorithms into results from conformal prediction. This conversion involves a calibration of the output for each compound and each label (class) in the evaluation set in relation to the output predictions for the compounds of the corresponding class in the calibration set.

A CP binary classification problem can have four possible outcomes. A test (evaluation) compound can be assigned a label for either of the two classes, assigned both labels (both classification) or none of the labels (empty classification). For a detailed description on how this calibration is performed, see Norinder and co-workers [40].

**Figure 1:**
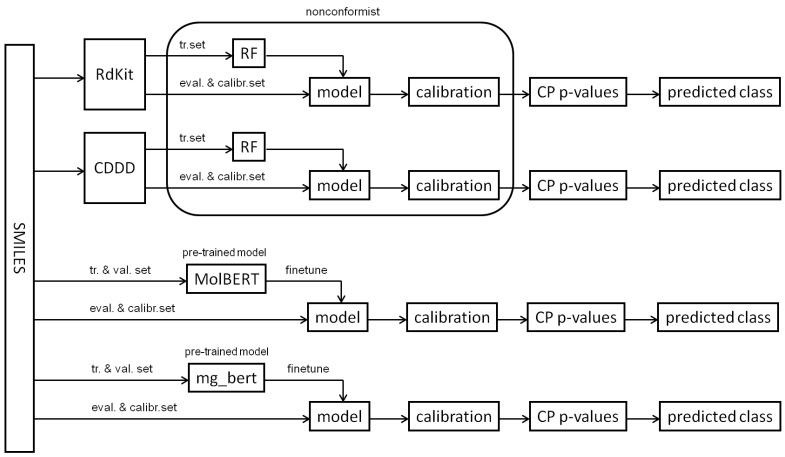
A flow chart overview depiction of the employed machine learning approaches. RdKit and CDDD = RdKit and CDDD descriptor calculation, tr. and val. set. = training and validation set, respectively, eval. and calibr. set. = evaluation and CP calibration set, respectively.

A flow chart overview of the employed machine learning approaches and calibration is depicted in Figure 1.

Validity and efficiency are two key measures in conformal prediction. Validity, for each class, is the percentage of correct predictions in Mondrian conformal prediction at a given significance level where the prediction contains the correct class. Thus, in binary classification the both classification is always correct (contains both available labels) while the empty classification is always erroneous (contains no labels). Models were considered valid when the resulting error rate does not exceed the set error rate (significance level) by more the 2.5%.

Efficiency, for each class, is defined as the percentage of single label predictions (only one label), regardless of whether the prediction is correct or not, at a given significance level. High efficiency is therefore desirable since a larger number of predictions are single label predictions and more informative as they contain only one class.

## 3. Results and Discussion

The aim of this study is to investigate how different molecular representations and algorithmic approaches may affect the predictive performance of the derived models. The present study therefore compares results from the traditionally used Random Forest/physico-chemical descriptor approach through an intermediate Random Forest/auto encoder representation to deep learning BERT/molecular-graph-based approaches.

**Figure 2:**
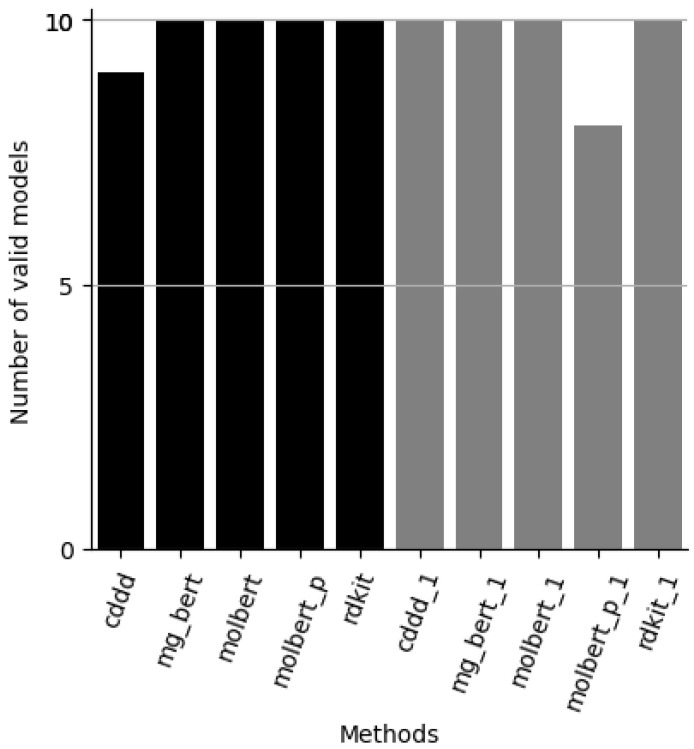
Number of valid evaluation set models (maximum 10) for each method type. Methods: cddd = RF/cddd 10 models, mg_bert = Molecular-graph-BERT/smiles 10 models, molbert = MolBERT/smiles 10 models, molbert_p = MolBERT/smiles 10 models with PubChem pre-trained model, rdkit = RF/rdkit 10 models, xxx_1 is the corresponding approach based on only 1 model.

**Figure 3:**
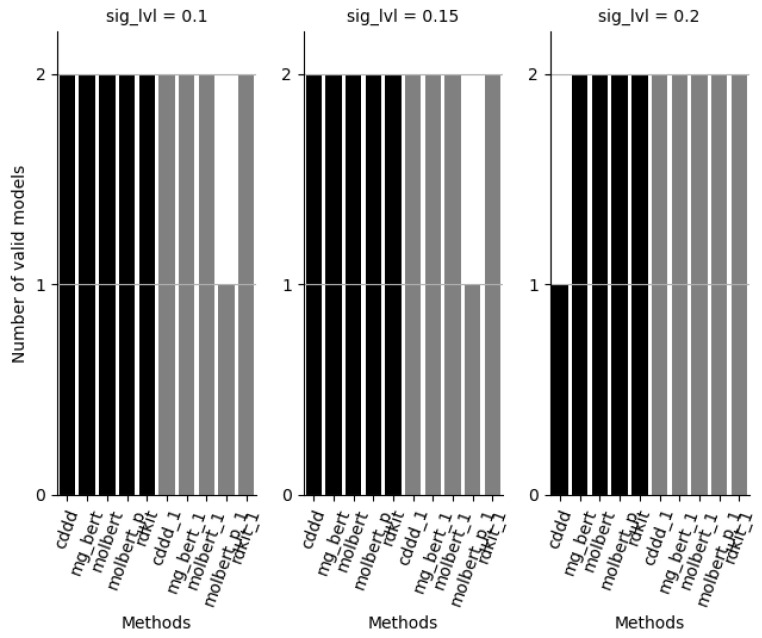
Number of valid evaluation set models, at significance levels 0.1, 0.15 and 0.2, for each method (maximum 2). Methods: cddd = RF/cddd 10 models, mg_bert = Molecular-graph-BERT/smiles 10 models, molbert = MolBERT/smiles 10 models, molbert_p = MolBERT/smiles 10 models with PubChem pre-trained model, rdkit = RF/rdkit 10 models, xxx_1 is the corresponding approach based on only 1 model.

**Figure 4:**
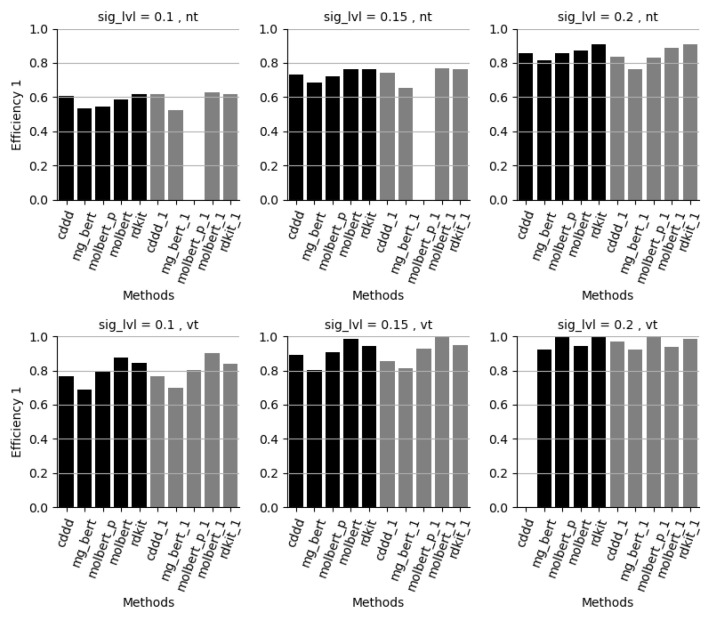
Evaluation set efficiency for class “1” for the 2 datasets (NT model upper row, VT model lower row), at significance levels 0.1–0.2, for each method. Class “1”: non-toxic class and very toxic class for the 2 datasets nt and vt, respectively. Methods: cddd = RF/cddd 10 models, mg_bert = Molecular-graph-BERT/smiles 10 models, molbert = MolBERT/smiles 10 models, molbert_p = MolBERT/smiles 10 models with PubChem pre-trained model, rdkit = RF/rdkit 10 models, xxx_1 is the corresponding approach based on only 1 model.

**Figure 5:**
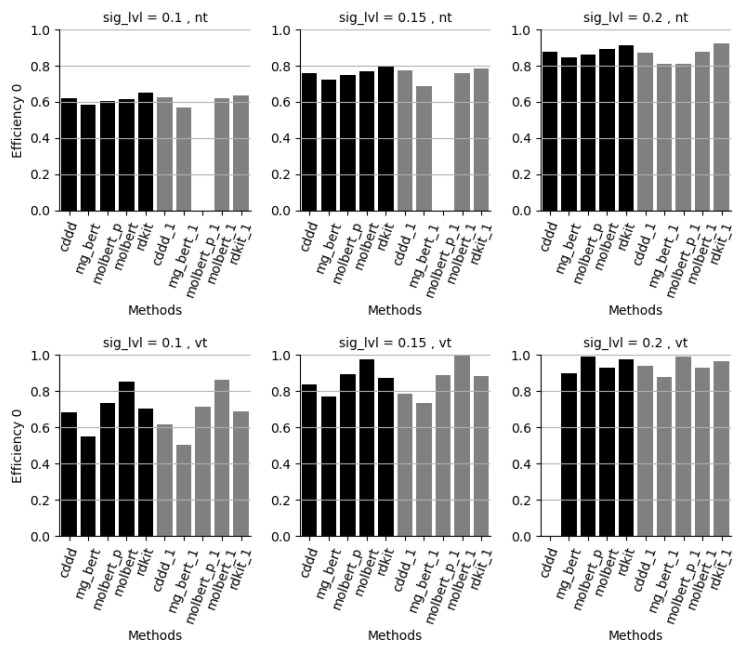
Evaluation set efficiency for class “0” for the 2 datasets (NT model upper row, VT model lower row), at significance levels 0.1–0.2, for each method. Class “0”: the other binary class for each dataset as compared to Figure 4. Methods: cddd = RF/cddd 10 models, mg_bert = Molecular-graph-BERT/smiles 10 models, molbert = MolBERT/smiles 10 models, molbert_p = MolBERT/smiles 10 models with PubChem pre-trained model, rdkit = RF/rdkit 10 models, xxx_1 is the corresponding approach based on only 1 model.

The results from the study are shown in Table 2 and Supplementary Materials Table S1 and depicted in Figure 2, Figure 3, Figure 4 and Figure 5.

From Figure 2, it can be noted that most methods, both ensemble and single models, produce valid models for significance level 0.1–0.3 with the exception of one CDDD ensemble model and two single MolBERT models based on the PubChem pre-trained model.

Figure 3 shows that both RDKit approaches and the BERT ensemble approaches as well as many single models result in valid models for both datasets at significance levels of primary interest, e.g., with error levels (significance levels) set at 10, 15 and 20%.

Figure 4 and Figure 5 show that the efficiencies, i.e., the percentage of single label predictions, for both class 1 and 0 at an acceptable error level of 10% are, on average, only 60–70%, which, for most cases, cannot be considered sufficient. At 15% acceptable error rate both the MolBERT and RDKit approaches show efficiencies for both class 1 and 0 close to or above 80% which ensures a large portion of single-label predictions. The efficiency is further increased at 20% acceptable error rate where all approaches have efficiencies well above 80% for both class 1 and 0. The performance is generally somewhat better for the catmos_vt dataset compared to the catmos_nt dataset.

The class distribution (class “0”/class “1”) is ~1.6:1 for catmos_nt and ~11:1 for catmos_vt which means that the former data set is rather balanced while the latter is rather unbalanced. This imbalance may, in turn, cause some issues for ML algorithms to properly handle the minority class [41,42,43].

From the catmos_nt results presented in Table 2 it can be noted that all of the developed models in this study performs similarly with respect to the SE/SP balance with an absolute average difference of 0.039.

The catmos_vt results presented in Table 2 show the graceful handling of the class imbalance in this dataset, by an absolute average difference between SP and SE for the four models (cddd not valid for class 1) in this investigation of 0.019, by using Mondrian conformal prediction. Furthermore, this balanced performance was the result of running the Mondrian CP framework in default mode and without the use of over- and/or under-sampling, weighting of classes or cost-sensitive measures.

The more balanced results from this investigation with respect to SE and SP are due to the independent handling of the two classes as part of the Mondrian conformal prediction calibration procedure; see Section 2.5 for more details.

The well-balanced SE/SP performance is of importance from the point of safety, i.e., not to err on the false negative side, when predicting a toxic compound to be non-toxic for the catmos_vt model. It is less of a problem for the catmos_nt model if a few more non-toxic compounds are predicted to be toxic from a safety perspective.

All methods in Table 2 are performing equally well on the balanced catmos_nt dataset while MolBERT, based on the larger pre-trained model (method “molbert” in Table 2), seems to be performing somewhat better than the other methods for the catmos_vt dataset with respect to SE and SP and the balance between them based on BA results from the 10 individual models (95% confidence, Mann–Whitney U test with Bonferroni correction for multiple testing) constituting the molbert ensemble results.

Advantages of the BERT approaches over the RDKit descriptor-based approach is that they are descriptor-free in that SMILES are used as input without the need for explicit descriptor generation prior to modeling and that the results from the models can be projected back onto the atoms of the molecules through their attention mechanisms. The advantage of the RDKit and CDDD approaches is shorter computation costs and that smaller datasets can be modeled with acceptable outcomes compared to using deep learning in the form of BERT that usually requires larger training sets.

## 4. Conclusions

The results show for the binary CATMoS non-toxic, almost equally balanced dataset that all methods perform equally well. The results also show that the MolBERT model based on a larger pre-trained network performs somewhat better compared to the rest for the binary CATMoS very-toxic dataset with an 11-fold difference between the two classes. The descriptor-free, SMILES based, deep learning BERT architectures seem capable of producing well-balanced predictive models with defined applicability domains. This work also demonstrates that the class imbalance problem is gracefully handled through the use of Mondrian conformal prediction without the use of over- and/or under-sampling, weighting of classes or cost-sensitive methods.

## Figures and Tables

**Table 1:** Number of compounds in each of the sets.

Dataset	Training Set	DL Validation Set	Evaluation Set	CP Calibration Set ^a^
catmos_nt	6004	662	2776	1670
catmos_vt	6449	717	2985	1789

^a^: Conformal prediction calibration set.

**Table 2:** Aggregated conformal prediction results at significance level 0.2.

Dataset	Method ^a^	Significance Level ^b^	Validity Minority Class 1	Validity Majority Class 0	Efficiency Minority Class 1	Efficiency Majority Class 0	Sensitivity (SE)	Specificity (SP)	Balanced Accuracy (BA)
catmos_nt	cddd	0.2	0.802	0.824	0.855	0.879	0.769	0.800	0.785
catmos_nt	mg_bert	0.2	0.798	0.848	0.814	0.845	0.751	0.821	0.786
catmos_nt	molbert_p	0.2	0.791	0.830	0.856	0.864	0.756	0.803	0.779
catmos_nt	molbert	0.2	0.797	0.830	0.873	0.892	0.767	0.810	0.788
catmos_nt	rdkit	0.2	0.800	0.805	0.909	0.912	0.780	0.786	0.783
catmos_vt	cddd	0.2	0.770	0.817					
catmos_vt	mg_bert	0.2	0.843	0.829	0.923	0.900	0.830	0.810	0.820
catmos_vt	molbert_p	0.2	0.798	0.819	0.996	0.991	0.798	0.820	0.809
catmos_vt	molbert	0.2	0.815	0.818	0.944	0.931	0.863	0.878	0.871
catmos_vt	rdkit	0.2	0.819	0.821	0.996	0.979	0.822	0.839	0.830

^a^: cddd = RF/cddd 10 models, mg_bert = Molecular-graph-BERT/smiles 10 models, molbert = MolBERT/smiles 10 models, molbert_p = MolBERT/smiles 10 models with PubChem pre-trained model, rdkit = RF/rdkit 10 models, ^b^ = CP significance level.

## Data Availability

The data used in this study are publicly available in reference [29].

## Supplementary Materials

The following supporting information can be downloaded at: https://www.mdpi.com/article/10.3390/molecules28010217/s1, Table S1: Conformal predictions results at significance levels 0.1–0.3.; list of calculated RDKit descriptors Materials S2: list of calculated RDKit descriptors.
