# Supplementary material for: Traditional Machine and Deep Learning for Predicting Toxicity Endpoints
Source: Molecules. 2022 Dec 26;28(1):217. doi: 10.3390/molecules28010217 (PMC9822478; doi:10.3390/molecules28010217)
Supplement: Supplementary file 1 [file molecules-28-00217-s001.zip › List of calculated RDKit descriptors S2.pdf]

List of calculated RDKit descriptors

RDKit descriptor	RDKit descriptor	RDKit descriptor
Chi0	Chi32	SMR_VSA10
Chi1	NHOHCount	SMR_VSA2
Chi2	NOCount	SMR_VSA3
Chi3	NumAliphaticCarbocycles	SMR_VSA4
Chi4	NumAliphaticHeterocycles	SMR_VSA5
Chi5	NumAliphaticRings	SMR_VSA6
Chi6	NumAromaticCarbocycles	SMR_VSA7
Chi7	NumAromaticHeterocycles	SMR_VSA8
Chi8	NumAromaticRings	SMR_VSA9
Chi9	NumHAcceptors	SlogP_VSA1
Chi10	NumHDonors	SlogP_VSA10
Chi11	NumHeteroatoms	SlogP_VSA11
Chi12	NumRotatableBonds	SlogP_VSA12
Chi13	NumSaturatedCarbocycles	SlogP_VSA2
Chi14	NumSaturatedHeterocycles	SlogP_VSA3
Chi15	NumSaturatedRings	SlogP_VSA4
Chi16	PEOE_VSA1	SlogP_VSA5
Chi17	PEOE_VSA10	SlogP_VSA6
Chi18	PEOE_VSA11	SlogP_VSA7
Chi19	PEOE_VSA12	SlogP_VSA8
Chi20	PEOE_VSA13	SlogP_VSA9
Chi21	PEOE_VSA14	TPSA
Chi22	PEOE_VSA2	VSA_EState1
Chi23	PEOE_VSA3	VSA_EState10
Chi24	PEOE_VSA4	VSA_EState2
Chi25	PEOE_VSA5	VSA_EState3
Chi26	PEOE_VSA6	VSA_EState4
Chi27	PEOE_VSA7	VSA_EState5
Chi28	PEOE_VSA8	VSA_EState6
Chi29	PEOE_VSA9	VSA_EState7
Chi30	RingCount	VSA_EState8
Chi31	SMR_VSA1	VSA_EState9
