# Supplementary material for: Traditional Machine and Deep Learning for Predicting Toxicity Endpoints
Source: Molecules. 2022 Dec 26;28(1):217. doi: 10.3390/molecules28010217 (PMC9822478; doi:10.3390/molecules28010217)
Supplement: Supplementary file 1 [file molecules-28-00217-s001.zip › Table S1. Conformal predictions results at significance levels 0.1 - 0.3.pdf]

Table S1. Conformal predictions results at significance levels 0.1 - 0.3.

dataset	method	signiflevel	validity1	validity0	efficiency1	efficiency0	SE	SP	BA
catmos_nt	cddd	0.1	0.911	0.913	0.609	0.619	0.854	0.859	0.856
catmos_nt	cddd	0.15	0.861	0.875	0.731	0.756	0.811	0.834	0.822
catmos_nt	cddd	0.2	0.802	0.824	0.855	0.879	0.769	0.800	0.785
catmos_nt	cddd	0.25	0.741	0.787	0.971	0.977	0.733	0.782	0.757
catmos_nt	cddd	0.3	0.675	0.733	0.907	0.928	0.744	0.790	0.767
catmos_nt	mg_bert	0.1	0.904	0.927	0.536	0.583	0.820	0.875	0.848
catmos_nt	mg_bert	0.15	0.857	0.889	0.685	0.721	0.791	0.847	0.819
catmos_nt	mg_bert	0.2	0.798	0.848	0.814	0.845	0.751	0.821	0.786
catmos_nt	mg_bert	0.25	0.749	0.795	0.939	0.954	0.733	0.785	0.759
catmos_nt	mg_bert	0.3	0.698	0.755	0.959	0.961	0.728	0.784	0.756
catmos_nt	molbert	0.1	0.902	0.937	0.587	0.616	0.833	0.898	0.865
catmos_nt	molbert	0.15	0.840	0.883	0.763	0.769	0.790	0.849	0.819
catmos_nt	molbert	0.2	0.797	0.830	0.873	0.892	0.767	0.810	0.788
catmos_nt	molbert	0.25	0.734	0.783	0.985	0.984	0.735	0.782	0.758
catmos_nt	molbert	0.3	0.684	0.737	0.890	0.919	0.769	0.801	0.785
catmos_nt	molbert_p	0.1	0.897	0.936	0.547	0.606	0.812	0.895	0.853
catmos_nt	molbert_p	0.15	0.844	0.885	0.721	0.746	0.783	0.845	0.814
catmos_nt	molbert_p	0.2	0.791	0.830	0.856	0.864	0.756	0.803	0.779
catmos_nt	molbert_p	0.25	0.747	0.782	0.969	0.967	0.739	0.775	0.757
catmos_nt	molbert_p	0.3	0.688	0.726	0.929	0.927	0.741	0.783	0.762
catmos_nt	rdkit	0.1	0.905	0.913	0.619	0.653	0.847	0.867	0.857
catmos_nt	rdkit	0.15	0.859	0.863	0.763	0.794	0.816	0.827	0.821
catmos_nt	rdkit	0.2	0.800	0.805	0.909	0.912	0.780	0.786	0.783
catmos_nt	rdkit	0.25	0.747	0.760	0.985	0.989	0.758	0.769	0.763
catmos_nt	rdkit	0.3	0.709	0.709	0.903	0.901	0.785	0.786	0.786
catmos_nt	cddd_1	0.1	0.900	0.903	0.617	0.624	0.838	0.845	0.841
catmos_nt	cddd_1	0.15	0.845	0.857	0.741	0.775	0.790	0.816	0.803
catmos_nt	cddd_1	0.2	0.809	0.820	0.836	0.873	0.772	0.793	0.782
catmos_nt	cddd_1	0.25	0.749	0.785	0.950	0.962	0.736	0.776	0.756
catmos_nt	cddd_1	0.3	0.694	0.746	0.945	0.959	0.734	0.778	0.756
catmos_nt	mg_bert_1	0.1	0.908	0.911	0.525	0.567	0.825	0.843	0.834
catmos_nt	mg_bert_1	0.15	0.870	0.866	0.654	0.687	0.801	0.805	0.803
catmos_nt	mg_bert_1	0.2	0.814	0.837	0.764	0.811	0.756	0.799	0.777
catmos_nt	mg_bert_1	0.25	0.757	0.792	0.885	0.923	0.725	0.774	0.749
catmos_nt	mg_bert_1	0.3	0.690	0.756	0.979	0.989	0.705	0.764	0.734
catmos_nt	molbert_1	0.1	0.877	0.912	0.626	0.619	0.804	0.857	0.831
catmos_nt	molbert_1	0.15	0.826	0.859	0.767	0.761	0.773	0.814	0.794
catmos_nt	molbert_1	0.2	0.779	0.804	0.890	0.879	0.752	0.778	0.765
catmos_nt	molbert_1	0.25	0.741	0.759	0.970	0.975	0.733	0.753	0.743
catmos_nt	molbert_1	0.3	0.692	0.720	0.940	0.941	0.736	0.765	0.751
catmos_nt	molbert_p_1	0.1	0.870	0.917					
catmos_nt	molbert_p_1	0.15	0.818	0.876					
catmos_nt	molbert_p_1	0.2	0.785	0.837	0.829	0.812	0.740	0.800	0.770
catmos_nt	molbert_p_1	0.25	0.742	0.790	0.934	0.912	0.723	0.769	0.746
catmos_nt	molbert_p_1	0.3	0.694	0.737	0.980	0.962	0.708	0.766	0.737
catmos_nt	rdkit_1	0.1	0.906	0.908	0.619	0.638	0.849	0.856	0.852
catmos_nt	rdkit_1	0.15	0.858	0.860	0.764	0.785	0.814	0.821	0.817
catmos_nt	rdkit_1	0.2	0.797	0.799	0.907	0.922	0.776	0.782	0.779
catmos_nt	rdkit_1	0.25	0.743	0.748	0.987	0.982	0.753	0.762	0.758
catmos_nt	rdkit_1	0.3	0.694	0.712	0.892	0.901	0.778	0.791	0.785

catmos_vt	cddd	0.1	0.891	0.917	0.766	0.683	0.858	0.879	0.868
catmos_vt	cddd	0.15	0.835	0.865	0.891	0.839	0.814	0.839	0.827
catmos_vt	cddd	0.2	0.770	0.817					
catmos_vt	cddd	0.25	0.734	0.768	0.944	0.913	0.778	0.840	0.809
catmos_vt	cddd	0.3	0.685	0.721	0.859	0.818	0.798	0.881	0.840
catmos_vt	mg_bert	0.1	0.931	0.911	0.690	0.552	0.901	0.838	0.870
catmos_vt	mg_bert	0.15	0.895	0.875	0.802	0.770	0.869	0.837	0.853
catmos_vt	mg_bert	0.2	0.843	0.829	0.923	0.900	0.830	0.810	0.820
catmos_vt	mg_bert	0.25	0.778	0.783	0.956	0.965	0.806	0.807	0.806
catmos_vt	mg_bert	0.3	0.710	0.722	0.859	0.858	0.826	0.842	0.834
catmos_vt	molbert	0.1	0.895	0.904	0.875	0.852	0.880	0.887	0.884
catmos_vt	molbert	0.15	0.855	0.861	0.984	0.976	0.852	0.858	0.855
catmos_vt	molbert	0.2	0.815	0.818	0.944	0.931	0.863	0.878	0.871
catmos_vt	molbert	0.25	0.734	0.768	0.839	0.851	0.875	0.903	0.889
catmos_vt	molbert	0.3	0.694	0.719	0.786	0.788	0.882	0.912	0.897
catmos_vt	molbert_p	0.1	0.915	0.908	0.794	0.737	0.893	0.876	0.884
catmos_vt	molbert_p	0.15	0.847	0.866	0.907	0.892	0.831	0.850	0.841
catmos_vt	molbert_p	0.2	0.798	0.819	0.996	0.991	0.798	0.820	0.809
catmos_vt	molbert_p	0.25	0.758	0.767	0.919	0.899	0.825	0.853	0.839
catmos_vt	molbert_p	0.3	0.718	0.724	0.851	0.825	0.844	0.877	0.860
catmos_vt	rdkit	0.1	0.903	0.911	0.843	0.706	0.885	0.874	0.880
catmos_vt	rdkit	0.15	0.855	0.868	0.944	0.874	0.846	0.849	0.847
catmos_vt	rdkit	0.2	0.819	0.821	0.996	0.979	0.822	0.839	0.830
catmos_vt	rdkit	0.25	0.778	0.775	0.931	0.884	0.836	0.876	0.856
catmos_vt	rdkit	0.3	0.722	0.724	0.863	0.804	0.836	0.901	0.869
catmos_vt	cddd_1	0.1	0.891	0.904	0.766	0.616	0.858	0.844	0.851
catmos_vt	cddd_1	0.15	0.871	0.851	0.855	0.784	0.849	0.810	0.830
catmos_vt	cddd_1	0.2	0.802	0.797	0.968	0.942	0.796	0.785	0.790
catmos_vt	cddd_1	0.25	0.730	0.747	0.935	0.909	0.780	0.822	0.801
catmos_vt	cddd_1	0.3	0.690	0.703	0.883	0.813	0.781	0.865	0.823
catmos_vt	mg_bert_1	0.1	0.911	0.891	0.698	0.505	0.873	0.784	0.829
catmos_vt	mg_bert_1	0.15	0.867	0.851	0.815	0.735	0.837	0.797	0.817
catmos_vt	mg_bert_1	0.2	0.827	0.798	0.923	0.877	0.812	0.770	0.791
catmos_vt	mg_bert_1	0.25	0.778	0.750	1.000	0.997	0.778	0.750	0.764
catmos_vt	mg_bert_1	0.3	0.726	0.700	0.911	0.890	0.796	0.787	0.792
catmos_vt	molbert_1	0.1	0.887	0.895	0.899	0.866	0.874	0.878	0.876
catmos_vt	molbert_1	0.15	0.871	0.841	0.996	0.995	0.870	0.841	0.856
catmos_vt	molbert_1	0.2	0.823	0.801	0.940	0.930	0.876	0.862	0.869
catmos_vt	molbert_1	0.25	0.742	0.747	0.851	0.826	0.872	0.904	0.888
catmos_vt	molbert_1	0.3	0.710	0.704	0.798	0.772	0.889	0.912	0.901
catmos_vt	molbert_p_1	0.1	0.883	0.897	0.802	0.716	0.854	0.856	0.855
catmos_vt	molbert_p_1	0.15	0.831	0.847	0.927	0.889	0.817	0.828	0.823
catmos_vt	molbert_p_1	0.2	0.794	0.806	0.996	0.990	0.794	0.804	0.799
catmos_vt	molbert_p_1	0.25	0.758	0.754	0.931	0.900	0.814	0.838	0.826
catmos_vt	molbert_p_1	0.3	0.685	0.708	0.843	0.813	0.813	0.871	0.842
catmos_vt	rdkit_1	0.1	0.895	0.897	0.839	0.690	0.875	0.851	0.863
catmos_vt	rdkit_1	0.15	0.847	0.847	0.948	0.886	0.838	0.828	0.833
catmos_vt	rdkit_1	0.2	0.810	0.802	0.984	0.967	0.824	0.829	0.826
catmos_vt	rdkit_1	0.25	0.754	0.755	0.915	0.870	0.824	0.868	0.846
catmos_vt	rdkit_1	0.3	0.685	0.705	0.839	0.789	0.817	0.894	0.856

Class “1”: non-toxic class and very toxic class for the 2 datasets nt and vt, respectively. Class “0”: the other binary class for each dataset. Methods: cddd = RF/cddd 10 models, mg_bert = Molecular-graph-BERT/smiles 10 models, molbert = MolBERT/smiles 10 models, molbert_p = MolBERT/smiles 10 models with PubChem pretrained model, rdkit = RF/rdkit 10 models, xxx_1 is the corresponding approach based on only 1 model. SE = Sensitivity, SP = Specificity, BA = Balanced accuracy.
